# Does macro-level general trust influence second language communication?: targeting prefectural capitals

**DOI:** 10.1186/s13104-023-06484-y

**Published:** 2023-09-11

**Authors:** Takehiko Ito, Hideo Ueichi

**Affiliations:** 1 Hosei University, Machida, Japan; 2 University of Tsukuba, Tsukuba, Japan

**Keywords:** General trust, Willingness to communicate, Multilevel analysis

## Abstract

**Objectives:**

This study investigated the macro-level effects of general trust on second language willingness to communicate (WTC) among Japanese individuals residing in 20 prefectural capitals. Previous research has shown the impact of individual-level general trust on WTC; however, macro-level effects have not been explored.

**Results:**

Multilevel analysis in this research revealed that individual- and prefectural capital-level general trust had a positive influence on WTC in English for Japanese people. Moreover, macro-level effects had a strong predictive power for WTC, thus, highlighting the potential of general trust to facilitate positive connections between people from different cultures.

## Introduction

The willingness to communicate (WTC) has strong predictive power for second language (L2) communication behavior, according to MacIntyre et al. [1]. They defined L2 WTC as the readiness to initiate a conversation in a second language with a specific person. Ito [2] demonstrated that general trust, which is the perception of how much people trust others [3], had a positive impact on the WTC in English among university students and the general Japanese population. This study suggests that general psychological tendencies can predict human language behaviors.

From a socio-ecological perspective, which shows that the human mind is formed by the social environment, Ito [4] investigated the impact of area-level relational mobility, which measures the degree to which people can meet new individuals [5], on the WTC in English. The study was conducted on Japanese individuals living in 20 prefectural capitals (typical cities). Multilevel analysis indicated that individual-level relational mobility had a positive effect on individual-level WTC in English. Moreover, prefectural capital-level relational mobility had a positive impact on prefectural capital-level WTC. This study suggests that human language behavior is influenced by social environment. This finding is consistent with Peng and Woodrow [6], who introduced a socio-ecological framework for WTC where teacher support, student cohesiveness, and task orientation had positive effects on WTC in English.

Ito [2] investigated the impact of individual-level general trust on language attitudes. Yamagishi [7] posits that social environments, such as relational mobility, shape the psychological tendency of general trust. If general trust is influenced by the social environment, then environmental-level general trust will have a positive effect on L2 WTC. However, no prior studies have examined this effect, and the present study aims to investigate the macro-level impact. Through this research, we examined how the social environment influences the human mind, which socio-ecological theory often reveals.

Ito [4] found that the level of relational mobility varied across prefectures. As previously mentioned, relational mobility shapes general trust [7]. Thus, the current study focuses on environmental-level general trust. We hypothesized that individual-level general trust would have a positive impact on the WTC in English, which replicates the findings of Ito [2]. Additionally, we hypothesized that prefecture-level general trust would have a positive effect on prefecture-level L2 WTC. This study investigated the impact of macro-level general trust on WTC. Macro-level general trust refers to general trust at the social-environmental level, where the level of general trust represents an aggregation of individuals’ trust within that environment.

Multilevel analysis was utilized to examine the macro-level effects. This method simultaneously analyzes individuals and groups hierarchically, and estimates the fixed effects of individuals and the random effects of groups [8]. This analysis examined the relationship between prefectural capital-level general trust and WTC, controlling for the impact of individual-level general trust on WTC. This mirrors the functionality of the multiple regression analysis. The present study targeted Japanese individuals living in each prefectural capital, while controlling for the variance in general trust within each prefecture.

## Method

### Participants

The study sample consisted of 2,240 Japanese individuals aged 30–40 years (1,115 men and 1,125 women; *M* = 31.52, *SD* = 5.26). All the participants lived in one of the 20 prefectural capitals and had previously participated in a study conducted by Ito [4]. The participants were individuals registered with an online survey company and residing in the capital of each prefecture. Specifically, we targeted individuals residing in the prefectural capital to control for variance in general trust within each prefecture.

### Procedure

An online survey was conducted from January 6 to 11, 2022. The survey links were delivered to the participants by the survey company and they accessed the questionnaire through personal computers or smartphones. Participants received monetary compensation after completing the survey.

### Questionnaire

#### WTC in English

The willingness to communicate scale was originally developed by McCroskey [9], and the Japanese version of the scale developed by Yashima [10] was used in this study. Participants were asked to rate their willingness to communicate on a 5-point Likert scale ranging from 1 (never) to 5 (strongly) across the 12 communication contexts. The scale included items such as “Talk in a large meeting of friends,” “Talk with an acquaintance,” and “Talk in a small group of strangers.” The scale had high internal consistency, with an alpha coefficient of 0.97.

#### General trust

The general trust scale was originally developed by Yamagishi and Yamagishi [11]; the Japanese version of the scale developed by Yamagishi [7] was used in this study. Participants were asked to rate their level of agreement with six items related to general trust on a 5-point Likert scale ranging from 1 (strongly disagree) to 5 (strongly agree). The scale included items such as “Most people are basically honest,” “Most people are trustworthy,” and “Most people are basically good and kind.” The scale had high internal consistency with an alpha coefficient of 0.84.

#### Big five personality traits

The “Ten Item Personality Inventory” scale was originally developed by Gosling et al. [12], and the Japanese version developed by Oshio et al. [13] was used in this study. It measured the five personality factors with two items each—extraversion (*r* = .31, *p* < .01), conscientiousness (*r* = .27, *p* < .01), neuroticism (reversed, *r* = .24, *p* < .01), openness (*r* = .19, *p* < .01), and agreeableness (*r* = .19, *p* < .01) [14], with responses ranging from 1 (strongly disagree) to 5 (strongly agree).

#### Chance of communicating with English speakers

For the item “How often do you talk with English speakers in your daily life?” responses were recorded on a five-point scale of 1 (not at all), 2 (once a month), 3 (once a week), 4 (three times a week), and 5 (every day). The answers were recorded as dummy variables as follows: 0 (participants who answered 1) and 1 (participants who answered between 2 and 5).

## Results

Table 1 presents the mean, standard deviation, and correlation matrix. The results indicate that general trust was positively correlated with WTC in English (*r* = .20, *p* < .01), which is consistent with Ito’s [2] findings on the positive impact of individual-level general trust on WTC in English. Additionally, the Big Five personality traits, except for agreeableness, were positively correlated with the WTC in English, replicating the results of studies 2 and 3 by Ito [2].

**Table 1:** Means, Standard Deviations, and Correlation Matrix

	*M*	*SD*	1		2		3		4		5		6		7		8		9	
1. WTC in English	2.26	0.99	―																	
2. General Trust	3.75	1.07	0.20	^**^	―															
3. Sex (M = 0, W = 1)	0.50	0.50	− .13	^**^	0.01		―													
4. Age	31.52	5.26	− .08	^**^	0.00		− .06	^**^	―											
5. Communicating Chance	1.47	0.87	0.33	^**^	0.07	^**^	− .08	^**^	− .05	^*^	―									
6. Extraversion	3.53	1.26	0.26	^**^	0.13	^**^	− .01		− .01		0.17	^**^	―							
7. Agreeableness	4.51	1.10	− .08	^**^	0.22	^**^	0.08	^**^	− .03		− .06	^**^	− .10	^**^	―					
8. Conscientiousness	3.66	1.15	0.15	^**^	0.09	^**^	− .04	^*^	0.05	^*^	0.10	^**^	0.26	^**^	0.14	^**^	―			
9. Neuroticism	3.58	1.15	0.20	^**^	0.18	^**^	− .19	^**^	0.04	^+^	0.10	^**^	0.26	^**^	0.17	^**^	0.32	^**^	―	
10. Openness	3.74	1.10	0.27	^**^	0.07	^**^	− .10	^**^	− .05	^*^	0.17	^**^	0.36	^**^	− .06	^**^	0.18	^**^	0.25	^**^

^**^p < .01, ^*^p < .05, ^+^*p* < .10

Table 2 shows the means, standard deviations, intraclass correlation coefficients (ICC), and 95% confidence intervals. The ICC represents the variance in scores between prefectural capitals [15]. The ICC for the WTC in English was 2% (*p* < .01), indicating that the WTC varied between prefectural capitals. Although the ICC for general trust was not significant (-0.1%, *n.s.*), the data had hierarchical structures, and multilevel analysis could be used [16]

**Table 2:** Means, Standard Deviations, ICC, and Confidence Intervals (95%)

	*M*	*SD*	*ICC*	Lower	Upper	*p value*
WTC	2.26	0.99	0.020	0.008	0.052	0.000
General Trust	3.75	1.07	− .001	− .004	0.008	0.584

After determining the ICC, we conducted a multilevel analysis. In this analysis, we employed grand-mean centering with general trust at the grand mean. According to Enders and Tofighi [17], centering at the grand mean (CGM) is suitable when the primary focus is on a Level-2 predictor and controlling for Level-1 covariates. Given that our research focused on the effects of group-level (Level 2) general trust on WTC, utilizing grand mean centering was appropriate. In line with Diez Roux [18], group differences stemming from group-level variables were considered contextual effects.

After controlling for individual-level factors such as sex, age, chance of communicating with English speakers, and the Big Five personality traits, individual-level general trust was found to have a positive influence on individual-level WTC in English (*b* = 0.15, *p* < .01; see Table 3). This finding is consistent with that of Ito [2], who found a positive relationship between individual-level general trust and WTC in English. In addition, after controlling for the individual-level factors, prefectural capital-level general trust had a positive impact on prefectural capital-level WTC in English (*b* = 1.14, *p* < .01). The prefectural capital level of general trust refers to the aggregation of individuals’ general trust in each prefectural capital. Conversely, individual-level general trust pertains to an individual’s general trust irrespective of the prefectural capital. These results indicate that the general psychological tendencies shared by residents in each prefectural capital had a stronger influence on WTC than individual-level general trust. This suggests that the level of trust within a community has a greater impact on the Japanese people’s willingness to communicate in English.

**Table 3:** The Effect of Prefectural Capital- and individual-level of General Trust on WTC

	*b*		*SE*	
Prefectural Capital Level				
Intercept	-1.99	+	1.11	
General Trust	1.14	**	0.30	
Individual Level				
General Trust	0.15	**	0.02	
Sex (M = 0, W = 1)	-0.16	**	0.04	
Age	-0.01	**	0.00	
Chance of Communicating	0.28	**	0.02	
Extraversion	0.09	**	0.02	
Agreeableness	-0.09	**	0.02	
Conscientiousness	0.04	*	0.02	
Neuroticism (reversed)	0.06	**	0.02	
Openness	0.11	**	0.02	

^**^p < .01, ^*^p < .05, ^+^*p* < .10

*b*, unstandardized coefficients

## Discussion

Correlation analysis revealed a positive correlation between general trust and WTC. This finding replicates Ito’s [2] study, which showed that individual-level general trust has a positive effect on WTC among Japanese people. Interestingly, almost all the Big Five personality traits showed a positive correlation with WTC. According to MacIntyre et al.’s [1] argument, personality traits form the basis of affective and cognitive variables in L2 language communication, which directly influence L2 WTC. The results of the present study confirm this psychological tendency.

The ICC in the present study showed the degree of difference in factor scores between groups, specifically in prefectural capitals. The significant ICC value for WTC indicate that attitudes towards second language communication varied between different areas, thus confirming the use of multilevel analysis. However, the ICC for general trust was not significant. The data structure consisted of samples from each prefecture nested within the Japanese population, which demonstrated the appropriateness of using a multilevel analysis owing to the hierarchical structure of the data.

The results of the multilevel analysis demonstrated that individual-level general trust had a positive influence on individual-level WTC in English, even after controlling for individual-level factors such as sex, age, chance of communicating with English speakers, and the Big Five personality traits. This finding is consistent with Ito’s [2] study, which showed the positive impact of individual-level general trust on WTC, even after controlling for the Big Five personality traits. These results suggest that individual general trust has a fundamental influence on WTC in the present study as well.

In addition, the analysis revealed that prefectural capital-level general trust had a positive impact on prefectural capital-level WTC in English even after controlling for all individual-level factors. This suggests that macro-level trust had an impact on positive attitudes towards English communication. Interestingly, the results showed that macro-level general trust had a greater impact than individual-level trust. These findings suggest that prefectures with high levels of general trust exhibit more positive behaviors towards second language speakers than prefectures with low levels of trust. Through this research, we examined how the social environment influences the human mind, which socio-ecological theory often reveals.

The present study focused on macro-level general psychological tendencies, which differs from Ito’s [2] study, which examined the impact of individual-level general trust on L2 WTC. General trust serves as a psychological factor that influences the initiation of second language communication. If this factor is shared within each environment, we can predict people’s general language behaviors in that specific environment.

Ito [4] found that prefectural capital-level relational mobility had a positive impact on prefectural capital-level WTC. However, researchers can use general trust as a simpler alternative for conducting social surveys. General trust scales typically consist of six items [7], whereas relational mobility scales consist of twelve items. The use of a smaller number of items may have increased the participants’ motivation to answer the questions.

In countries where English is a foreign language such as Japan, opportunities to communicate with English speakers are limited. The results of the present study showed that Japanese individuals had a low chance of communicating in English (*M* = 1.47, *SD* = 0.87). As stated earlier, general trust can be useful in predicting human language communication behaviors. High school or university students have limited opportunities to communicate with second language speakers; however, by examining their general psychological tendencies, such as general trust, interventions can be developed to promote second language communication. Moreover, this study revealed the macro-level effects of general trust on WTC. If educators create a classroom environment with higher levels of trust, members will have more positive attitudes towards English communication.

### Limitations

Regarding limitations, this study did not examine the interaction effects between general trust and social environments, such as relational mobility, on WTC. If general trust is influenced by social environments, its’ effects may also be influenced by them. Therefore, future studies should investigate the interaction effects between general trust and social environments to better understand their combined impact on WTC.

## Conclusion

This study examined the effects of general trust on English WTC for Japanese individuals at the individual- and prefectural capital-levels. Macro-level effects had a strong predictive power for WTC, highlighting the potential of general trust to facilitate positive connections between people from different cultures.

## Data Availability

The datasets used and analyzed during the current study are available from the corresponding author on reasonable request.

## List of abbreviations

ICC: Intraclass correlation coefficient
WTC: Willingness to communicate
